# Supplementary material for: Chromothripsis during telomere crisis is independent of NHEJ, and consistent with a replicative origin
Source: Genome Res. 2019 May;29(5):737–49. doi: 10.1101/gr.240705.118 (PMC6499312; doi:10.1101/gr.240705.118)
Supplement: Supplemental Material [file supp_gr.240705.118_Supplemental_file_1.zip › contigs/annotated_contigs/DB50/contig.2.DB50_length_773_mean_cov_10.6727037516.docx]

**DB50_length_773_mean_cov_10.6727037516**

```
TAGAAGGAGCACCTACCATGATCTGAGGGGGTGGAGTCAGGGAAGCTTCCCAGAAGAAGTGACATTTAAGCTGAGACCTGAAGGATGAG
 >chr17:28249018-28249334 + E=3e-175
TAGGAAATAGTTAGGTAGAGAGATGAGGGAAAAGTGTTCCAGGTAAAGGAAACAGCATGTGCAAAAGCCTAAAGACCAAAGGGAGCATG

GGCATGTCAGCTTAACTGTGAGCAGTGTGCAAGGCAGGGACTTGGGAAGAGACAGGGCTGAAGAGGTAAGCAAGGCTGGATCATTTAAG

GTCTCACTAGCAATTCGAATTATAGACTTTATTCTAAGAATAATACAA|A|TTTAGTGTCTTAGACCAGTCCCCTGAGACCTGTTCCTA
                                                 >chr17:28210737-28211195 + E=1e-260
TCAATTATCTCCTGTCTCACTTTTATCTTCTATCTCTTTCCCCACTGCCATTACTTTGGTTCATTTCCTTTAGCACAGAAACATTTTCA

AGTCTACTGCATACTTTAAAATCAAGAAAAATATTTGACCTTGCATTCCTCCATCTAGATATAATCCTTTTTCTCCTTCTATTCATGAG

AACTCTTGTGAAAATGAATACTCTATTTTTACTGTTTCTTTACATACATATATATATGATGAGTGAGAGAGGTTACTATGTTGCCCAGG

CTGGTCTCGAACTCCTGGGCTCAAGCAATCCTCCCACCTTGGCTTCCACAAGTGCTGGGATTACAGGTGTGAGCCACTACACCTGGCCT

ATATTCACAATTTGTTTGATCAATTTTCTCTTCAACTTACCTCAATCAGGCTTCTCTGTCCCA
```
